# What’s in the Sound? Common and Language-Specific Patterns in Brain Activation and Functional Connectivity for Phonological Awareness in Spanish–English Bilinguals

**DOI:** 10.1111/mbe.12410

**Published:** 2024-03-27

**Authors:** Nia Nickerson, Xin Sun, Valeria Caruso, Kehui Zhang, Chi-Lin Yu, Rachel Eggleston, Natasha Chaku, Xiaosu Hu, Teresa Satterfield, Ioulia Kovelman

**Affiliations:** 1 University of Michigan; 2 University of British Columbia; 3 Indiana University

## Abstract

Phonological awareness is the stepping-stone to learning to read as it helps children map language sounds onto letters. Theories of bilingualism posit that phonological awareness is a language-common literacy skill. However, bilingual learners are also thought to build language-specific representations. To illuminate common and specific dual-language processes, we asked bilingual Spanish–English heritage language speakers (*N* = 60, *M*_age_ = 8.2) to complete a phonological sound-matching task in Spanish and English during functional Near Infrared Neuroimaging (fNIRS). The left perisylvian activation was common across bilinguals’ two languages, including similar active regions and functional connections. The findings further revealed language-specific modulation of the system with more robust engagement of the temporal networks for Spanish and frontal networks for English. We interpret the results in the context of analytically demanding reading experiences in English and more informal home-based Spanish language experiences typical of heritage language speakers.

Phonological awareness (PA) helps children map language sounds onto print to support literacy development across languages (Ziegler & Goswami, 2005). Theories of bilingualism posit that learning to read in two languages likely involves the development of both shared and language-specific literacy skills (Cummins, 2017). Within bilinguals’ literacy systems, PA is generally considered one of the most common or otherwise cross-linguistically shared literacy skills (Chung, Chen, & Geva, 2019). At the same time, language experiences play pivotal roles in shaping bilingual literacy systems, including the quantity and quality of literacy instruction (Sun et al., 2022). The present work examines bilingual effects on the brain bases for PA in bilingual Spanish–English heritage language speakers.

## Heritage Language Bilinguals

Two types of childhood bilinguals are generally considered in neuroscience research: world language learners, who acquire a second language through formal schooling, and dual-language learners, or bilingual first language (L1) learners, who acquire two languages in immersive contexts. Within the bilingual L1 category, heritage language (HL) speakers are exposed to a home language, typically from birth, that is distinct from the community’s language (Ortiz-Villalobos, Kovelman, & Satterfield, 2024; Valdés, 2000).

World language learners exhibit stable distinctions between patterns of first language (L1) and second language (L2) proficiency and use (Gass & Glew, 2011). In contrast, heritage language (HL) bilinguals are believed to benefit from a language learning advantage because their bilingual experience falls within early periods of brain development for language function (Sulpizio, Del Maschio, Del Mauro, Fedeli, & Abutalebi, 2020; Weber-Fox & Neville, 2001). However, HL learners often face sociocultural and educational challenges to becoming bilingual in monolingual formal schooling contexts (Cummins, 2005; De Houwer, 2018). Our work examines the effects of dual-language proficiency on reading development in young bilingual HL speakers during the formation of the brain’s literacy networks.

## Neurobiology of Phonological Awareness

Understanding the neurobiological bases of PA has been a critical point of inquiry among developmental neuroscientists, given the skill’s central role in literacy development and its dysfunction in dyslexia, a life-long difficulty in reading and learning to read (Norton, Beach, & Gabrieli, 2015). PA skills are typically associated with the functionality of the dorsal neurocircuitry for language function, which includes the dorsal aspect of the left inferior frontal gyrus (dIFG), posterior superior temporal gyrus (STG), and the arcuate fasciculus white matter tract that connects these regions (Hickok & Poeppel, 2016). As children become older and better readers, activation along this neurocircuitry becomes more left-lateralized and focal, especially within the dIFG (Martin, Schurz, Kronbichler, & Richlan, 2015) and STG (Cao, Brennan, & Booth, 2015). Functional connectivity, or the statistical relationships of cerebral signals over time, between these regions, is also stronger in children with typical literacy development, especially when compared with children with dyslexia, who typically demonstrate deficits of phonological processing (Cao et al., 2017; Gaudet, Hüsser, Vannasing, & Gallagher, 2020; Norton et al., 2014).

## Neurobiology of Bilingualism and Phonological Awareness

Theories on bilingual literacy, such as the Interdependent Continuum Model (ICM), suggest that linguistic and orthographic experiences impact skill transfer between a bilingual’s two languages (Chung et al., 2019). PA is generally considered the most easily transferred literacy skill, as the operations involved in phonological awareness (e.g., the ability to recognize syllables, rhymes, and onset rhymes) are fundamentally similar across languages. Therefore, we predict that HL bilinguals will engage the dorsal or phonological language networks in both English and Spanish, including the left IFG and STG regions. At the same time, activation patterns may vary across the two languages as a function of orthographic characteristics or children’s experiences and proficiency in that language.

Cross-linguistically, sound-to-letter mapping principles are more transparent or consistent in Spanish than in English (Jasińska, Berens, Kovelman, & Petitto, 2017; Kovelman, Baker, & Petitto, 2008; Serrano et al., 2011). In a neuroimaging study of reading by Paulesu et al. (2000), monolingual speakers of Italian, a phonologically transparent language, showed greater activation in the left temporal regions, while monolingual speakers of English, a phonologically opaque language, showed greater activation of the left frontal and left inferior posterior temporal areas. The authors interpreted the results as reflecting greater automaticity of phonological processing in phonologically transparent language. Therefore, bilingual children may develop different processing patterns for each orthography (Das, Padakannaya, Pugh, & Singh, 2011).

Language proficiency also plays a key role in neural organization for dual-language function. For instance, an ERP study of adult Spanish–English heritage speakers found that neural response in English, the second language of acquisition but the dominant language of use during testing, supported greater automaticity during a lexical decision task (Ng & Wicha, 2013). For heritage language bilinguals, literacy proficiency tends to be stronger in the majority language, such as English in the US. A word-reading fMRI study by Hernandez, Woods, and Bradley (2015) observed that adult Spanish–English bilinguals had better English but similar Spanish reading abilities compared with bilingual children in the study. The adults also showed stronger left middle temporal gyrus (MTG) activation in English, attributed to literacy gains in the language, but more robust right MTG activation in Spanish, attributed to overall maturational gains. In sum, multiple factors, including education, patterns of daily language use, and proficiency, can affect how early-exposed bilinguals develop literacy skills and neural representations for those skills.

To uncover the influences of cross-linguistic differences and dual-language proficiency differences on the neural architecture for language in the bilingual brain, we ask: do bilingual Spanish–English children develop language-specific patterns of engaging the dorsal phonological network with stronger activation in the left frontal in English and left temporal regions in Spanish? Moreover, is proficiency in each language associated with activation strength across similar regions, or is it more specific to language-specific parts of the phonological network? Whereas much of the prior work addressed this question with adults (cited above), the innovation of this work is to examine children who are in the process of developing key language and literacy skills.

## Functional Connectivity

Neuroimaging studies on bilingual language and literacy development suggest that in early-exposed and high-proficiency bilinguals, the brain regions involved in language and literacy development are similar across the bilinguals’ two languages and similar to monolinguals (Brignoni-Perez, Jamal, & Eden, 2020; Brignoni-Pérez, Jamal, & Eden, 2022). Bilingualism research on early-exposed and high-proficiency bilinguals often examines whether these brain regions are active in the same way and exhibit similar or different communication patterns between the bilinguals’ two languages, possibly as a factor of dual-language experiences and proficiency. The current inquiry also includes a functional connectivity approach to better examine the dynamics by which perisylvian language regions communicate with each other across bilinguals’ two languages.

Functional connectivity research comparing children and adults finds that adults tend to exhibit stronger long-distance connections between frontotemporal language regions (e.g., IFG and Parietal or STG) and stronger connections between temporal and occipital regions (Lane & Gates, 2017). These developmental changes reflect functionality improvements in the neural pathways for processing language and connecting spoken and orthographic forms of language (Smith et al., 2011).

The effects of bilingualism on the development of these functional networks still need to be better understood. Of the few available and most relevant studies, Brignoni-Perez et al. (2020), Brignoni-Pérez et al., 2022 used fMRI to examine early-exposed, high-proficiency Spanish–English adult HL bilinguals as well as proficiency-matched English monolinguals. They found no significant differences in brain activity between bilinguals and monolinguals or between bilinguals’ two languages. Nevertheless, functional connectivity analyses revealed that bilinguals had stronger associations between the left STG and the left lingual gyrus and between the left middle frontal gyrus and the left anterior cingulate cortex. Monolinguals did not show any stronger connections than the bilinguals. The within-group bilingual cross-linguistic comparison showed stronger associations between the left middle occipital gyrus and bilateral lingual gyrus extending to the cerebellum, as well as a stronger association within parietal loci. The authors attributed stronger functional connectivity with the lingual gyrus and between the parietal regions in English to the greater visual complexity and phonological opacity of English orthography, as these regions are critical to visual and orthographic processing. In the present study, we extend the investigation of the effects of bilingualism on the development of functional language networks to young bilingual readers by examining functional connectivity patterns in the context of developmentally relevant PA tasks.

Among many available functional connectivity approaches, Group Iterative Multiple Model Estimation (or GIMME; Gates & Molenaar, 2012) is notable for its usefulness with intensive or dense data collection. It is a person-specific network-mapping technique used to uncover meaningful shared relations among a priori specified variables (e.g., ROIs) over relatively short periods. GIMME bridges nomothetic and idiographic approaches—it does so by using a data-driven algorithm (based or unified structural equation modeling or uSEMs) to uncover relations that are unique to an individual, shared by a subset of individuals, or common across all individuals (Beltz & Gates, 2017). In other words, GIMME computes the map of connections between ROIs at both the group and individual levels. It is thus well suited for uncovering similarities or differences among bilinguals’ language processing in Spanish and English. In a previous study, we found that children’s HL proficiency (i.e., in Spanish) contributed to overall network density (i.e., the proportion of connections in the network) as measured during the English morphological awareness task (Sun et al., 2022). We extend this inquiry to compare functional networks across bilinguals’ two languages during a PA task.

## The Present Study

Phonological awareness is a cornerstone of literacy success in alphabetic languages such as English and Spanish. Abundant information exists on the neural organization for PA in monolingual children with neurotypical development, as well as children with dyslexia (Martin et al., 2015; Norton et al., 2015). To our knowledge, however, few neuroimaging studies examine PA in emerging bilingual readers (Nickerson & Kovelman, 2022). The lack of research stems from the complexity of studying bilingual phenomena: children may vary greatly in their dual-language proficiency and contexts of learning across their two languages. Moreover, HL learners in the US are primarily racial minorities, most of whom are generally underrepresented in neuroimaging research (Webb, Etter, & Kwasa, 2022). Note that fNIRS neuroimaging in our study was carefully adjusted to accommodate children with different hair colors and textures.

The Interdependence Continuum model posits that bilingual children’s two languages interact within the same language system and that the following key parameters guide this interaction: (i) *saliency* or the extent to which a particular feature of language is more prevalent or of a higher statistical frequency in one of the two languages; (ii) *generalizability* vs. language-specificity of a given literacy skill; and (ii) *linguistic distance* between the two languages and their underlying spoken and orthographic structures. In Spanish, PA is generally considered more salient than in English as sound-to-letter mapping is more consistent in Spanish, thus likely creating a stronger transfer from Spanish to English than vice-versa. Furthermore, PA is considered one of the most language-general literacy skills, as, a common skill across languages is being able to compare words and decide if they start with the same sound. Finally, Spanish and English are Indo-European languages that share many phonological similarities, creating substantial opportunities for cross-linguistic transfer. This leads us to a prediction that bilingual speakers of Spanish and English likely form a highly reciprocal PA system to subserve each language. This is likely reflected in the engagement of similar brain regions at the neurological level.

Early-exposed bilinguals with high dual-language proficiency have been shown to activate the same brain regions during similar language tasks (Brignoni-Perez et al., 2020; Brignoni-Pérez et al., 2022). However, these same regions might be engaged differently in relation to the specific characteristics of the given language and bilinguals’ dual-language proficiency. Neuroimaging comparisons of monolingual English speakers and a language with higher phonological transparency (e.g., Italian, Spanish) find stronger engagements of frontal regions in English relative to more phonologically transparent languages (Paulesu et al., 2000). In these cases, frontal regions likely support complex analytical processes for sound-to-letter mappings, whereas temporal regions support the more automated sound-to-letter retrieval processes (Brignoni-Perez et al., 2020; Brignoni-Pérez et al., 2022). In English, bilingual readers may thus show stronger engagement and functional connectivity in left frontal regions. In contrast, in Spanish, they may show stronger engagement and functional connectivity in left temporal regions.

The study thus aimed to uncover children’s emerging neural architecture for dual-language phonological awareness. We hypothesized that bilingual Spanish–English speakers would exhibit stronger activation and functional connectivity in the left frontal regions in English and stronger activation and functional connectivity in Spanish, as previously found in monolinguals of these languages. We also explored the relation between participants’ proficiency and brain activity in each of their languages. We asked whether brain-behavior relations would be similar across bilinguals’ two languages in the context of cross-linguistic transfer and the generalized nature of PA skills or different due to either aforementioned cross-linguistic differences as well as differences in the manner in which heritage language learners are learning to read in their dominant language of instruction (English) and their heritage language (Spanish). This second question is exploratory as we only have one group of heritage language learners. The study thus aimed to provide an important step toward filling gaps in our understanding of the neurobiology of heritage language and literacy development.

## METHOD

### Participants

Sixty Spanish–English speaking children educated in Southeast Michigan, where Hispanic and Latinx families are an ethnic minority (∼5% of the area’s population; US Census, 2021), participated in the study (34 F, *M*_age_ = 8.2, SD = 1.4; see Table 1). The study was approved by the institutional review board at the University of Michigan, and families were paid for their participation. The data associated with this project are freely available at the Deep Blue repository and are accompanied by an extensively detailed manuscript by Sun et al. (2022), to which we refer throughout the methods section due to space limitations. Only participants with an overall fNIRS task accuracy above 65% were included in the analysis (participants’ fNIRS task performance was higher in English than in Spanish, likely because the participants attended English-only schools). For further information about the participants and experimental procedure, please see Supplementary Material 1, Participant section.

#### Procedure

After the initial screening protocol, participants came to our University of Michigan laboratory to complete behavioral and neuroimaging tasks. Parents and children completed consent and assent forms, respectively. Participants completed tasks in both languages during one visit to reduce attrition.

#### Behavioral Assessments

Participants completed behavioral measures of language and literacy in both Spanish and English including *receptive vocabulary* (PPVT-5: Dunn, 2019; TVIP: Dunn, Padilla, Lugo, & Dunn, 1986), *phonological awareness* (Elision subtests of Comprehensive Test of Phonological Processing (CTOPP-2): Wagner et al., 2013; Phonological Processing in Spanish (TOPPS): Francis et al., 2001), *reading comprehension* (Passage Comprehension, Woodcock Johnson: Schrank, McGrew, & Mather, 2014; Passage Comprehension, Bateria III Woodcock-Munoz: Muñoz-Sandoval, Woodcock, McGrew, & Mather, 2005) as well as *single word reading* (Letter-Word Identification, Woodcock Johnson (LWID): Schrank et al., 2014; Word Identification, Bateria III Woodcock-Munoz: Muñoz-Sandoval et al., 2005).

### fNIRS Phonology Task

Participants completed an auditory phonological awareness (PA) task in English and Spanish while undergoing fNIRS neuroimaging (Hu, Wagley, Rioboo, DaSilva, & Kovelman, 2020; Sun, Marks, Zhang, et al., 2022). In each trial, children heard three words. The first word was the target (e.g., *teeth*). The following two words matched the target either phonologically by starting with the same sound (e.g., *truth*), or semantically (e.g., *mouth*). Participants were required to press a button to indicate the phonological match. Participants were required to press a button to indicate the exact word match. The task included 48 trials in each language, presented in blocks of four 7.5 s trials with 12 blocks per language. Each trial started with the target word, followed by the 2nd word in about 1.5 s and the 3rd word in another 1.5 s; the remaining 3 s included decision and intertrial periods. Each block lasted 30 s, with six seconds of rest between blocks. The total duration of the task was approximately 7.2 min. Within each language, all stimuli were matched on word length (phoneme, syllables, letters), age of acquisition (Goh, Yap, & Chee, 2020), and frequency (COCA, Davies, 2020). The task has been fully described and validated with 343 children in Sun et al., 2022 reaching a reliability of *α* = .98.

### fNIRS Data Acquisition

Neuroimaging data were collected using the fNIRS TechEN-CW6 system using 23 channel configuration per hemisphere, designed to capture key language regions including frontal, temporal, and parietal areas. The probest was rigorously tested for anatomical localization, see Hu et al. (2020) and Sun, Marks, Zhang, et al. (2022) for fNIRS set-up detail, and Appendix Table 1 for channel anatomical localization information.

### fNIRS Data Processing

#### Preprocessing

fNIRS data were analyzed with the Nirs brain AnalyzIR, a Matlab-based toolbox (Santosa, Zhai, Fishburn, & Huppert, 2018), as well as in lab developed scripts. Preprocessing procedures are detailed within Sun, Marks, Zhang, et al. (2022).

#### Standard GLM Contrast Analyses

Data at the *individual level* were trimmed in length to keep only 5 s before and after the experimental task baseline. Data were then downsampled from 50 to 2 Hz, as the range of interest for fNIRS signal is within 0–1 Hz. The Modified Beer–Lambert Law then converted the optical density data to hemoglobin concentration data. Temporal and dispersion derivatives were added to the canonical hemodynamic response function (HRF), in addition to the discrete cosine transform (DCT) matrix to account for the signal drift over time. The General Linear Model (GLM) was used to analyze the individual level data and yielded individual level regression coefficients for oxygenated hemoglobin (HbO) and deoxygenated hemoglobin (HbR) signals for each channel. At the *group level*, two linear mixed effects models for each language were used following the results from individual level GLMs. Age was included as a covariate in each model. *Brain-behavior* correlations were performed using phonological awareness measures in each of the respective languages while also controlling for age. To be consistent across the GLM and GIMME analyses, the analytical contrasts were task > resting baseline.

#### Functional Connectivity Analyses

**Fig. 1.**
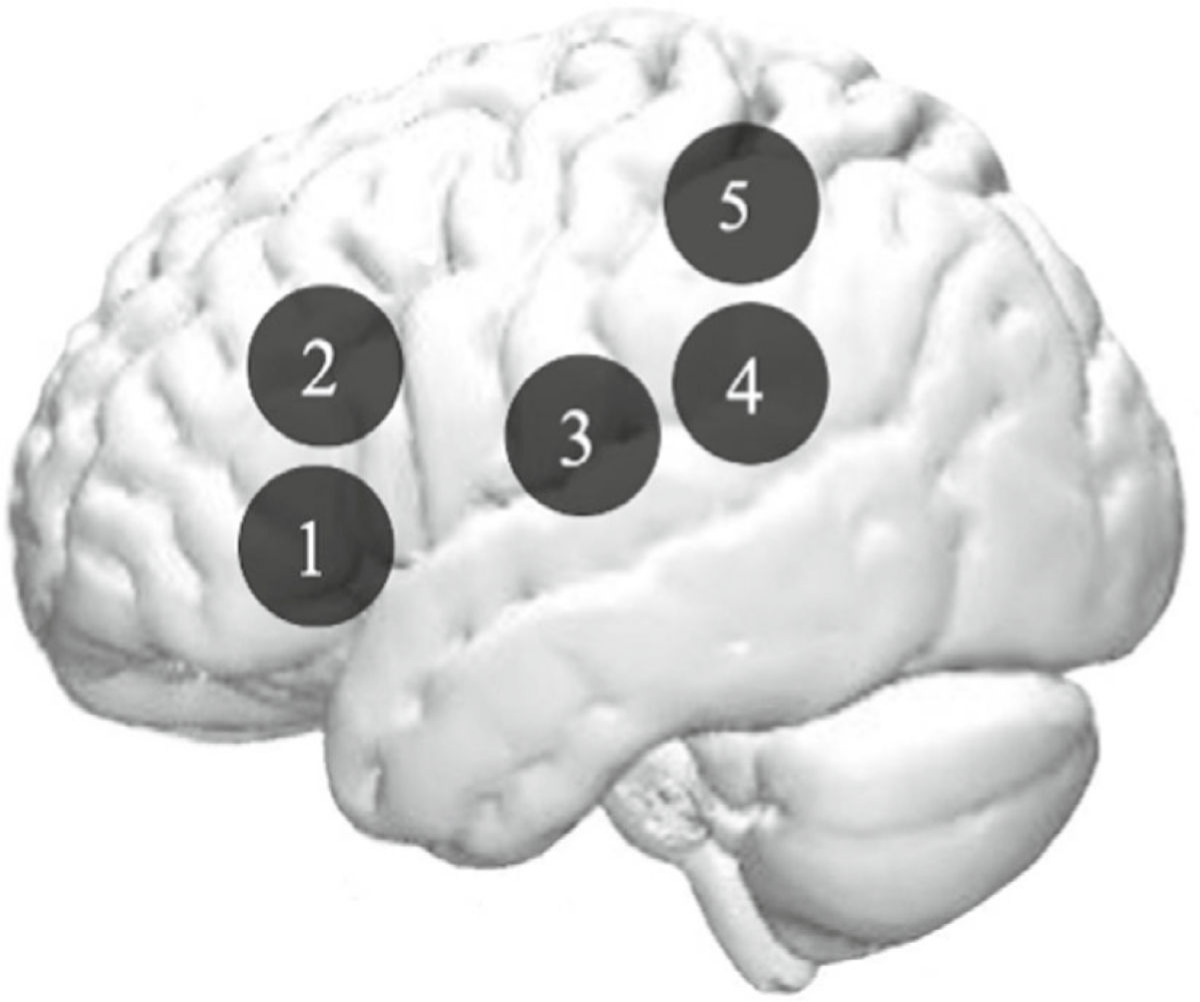
*GIMME ROIs*, GIMME variables displayed within the left hemisphere. IFG: Inferior Frontal Gyrus, MFG: Middle Frontal Gyrus, STG: Superior Temporal Gyrus, TTG: Transverse Temporal Gyrus, IPL: Inferior Parietal Lobule, L-Left R-Right. Only left hemisphere is displayed: Right 6 = left 3, right 7 = left 4.

**Fig. 2.**
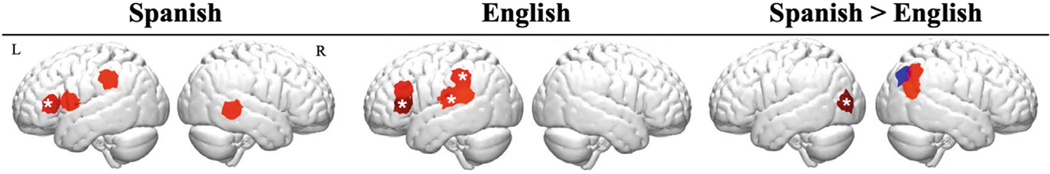
Bilinguals’ neural activation during the Spanish and English Phonology Task (task vs. rest contrasts). All channels are significant at *p* < .01; the channels with a * are also significant at the FDR corrected level of *q* < .05. In the direct cross-linguistic comparison, red denotes stronger activation for Spanish, and blue denotes stronger activation for English.

As appropriate for a within-group and repeated measures design, we use Confirmatory Subgroup GIMME. Confirmatory Subgroup GIMME (CS-GIMME, Henry et al., 2019) was used to construct ROI networks reflecting directed, functional connectivity maps for the 26 participants who had usable data in both languages (*N* = 26, *M*_age_ = 8.4, SD = 1.15). See Appendix Table 2 for more information about these participants. CS-GIMME uses unified structural equation models (uSEMs with a grouping algorithm to derive sparse network maps of directed relations among prespecified variables). ROIs selected for the GIMME are detailed in Figure 1. These seven ROIs were selected because they corresponded to the fNIRS channels that showed significant activation in the tasks (as indicated by the GLM analysis; see Figure 2 and Table 2). These ROIs are consistent with regions previously associated with phonological processing (e.g., Enge, Friederici, & Skeide, 2020). For each ROI, the oxy-hemoglobin (HbO) time-series data were obtained by applying the modified Beer–Lambert Law to optical density data down-sampled to 2 Hz (as recommended by Beltz & Gates, 2017).

GIMME analysis was performed with the CS-GIMME package in R (Chaku & Beltz, 2022; Lane & Gates, 2017, https://cran.rprooject.org/web/packages/gimme). Although GIMME estimates the directions of contemporaneous relations, these directions cannot be interpreted in causal or temporal terms (see Beltz & Gates, 2017 for further explication). Therefore, we report them in Figure 4, but we do not make causal claims about directionality. The effect of language on the connections estimated by GIMME at the group level was assessed with a mixed Analysis of Variance (ANOVA), with language (English, Spanish) and connections (GIMME resulted in two connections present for all participants in both languages) as within-participant factors, and participants modeled as a random effect. For more methodological detail, please see Supplementary Material 1, the GIMME section.

### Inclusive *fNIRS* Neuroimaging

fNIRS neuroimaging data quality can be impacted by skin color, hair color, texture, and thickness as signal relies on close skin contact (Parker & Ricard, 2022). Short, blonde hair allows for a reliable signal to be captured; but for participants with dark and thick hair, obtaining a reliable signal is much more effortful. Therefore, research groups such as ours dedicate much effort to building fNIRS set-ups that can achieve quality data for individuals with varied hair color and texture. Supplementary Material 1 details our culturally sensitive data collection methodology.

## RESULTS

### fNIRS Neuroimaging Results

#### Within and between Language Effects

Figure 2 reveals that the *Spanish* phonology task elicited activation in left IFG, Inferior Parietal (IPL), and right postcentral/MTG region. The *English* phonology task elicited activation in left IFG, left postcentral/STG, STG, and IPL. A direct comparison between the two languages revealed that the Spanish phonology task elicited stronger activation in bilateral temporal regions (left occipitotemporal and right STG and temporoparietal), whereas English elicited stronger right occipitoparietal activation. Sound-matching is one of the simplest and earliest acquired phonological manipulations; the levels of neural activation were relatively low even though they were considered in relation to the resting baseline. In Figure 2, results are shown at both corrected and uncorrected thresholds for readers’ consideration of the exploratory nature of the findings.

#### Brain Behavior Correlations

**Fig. 3.**
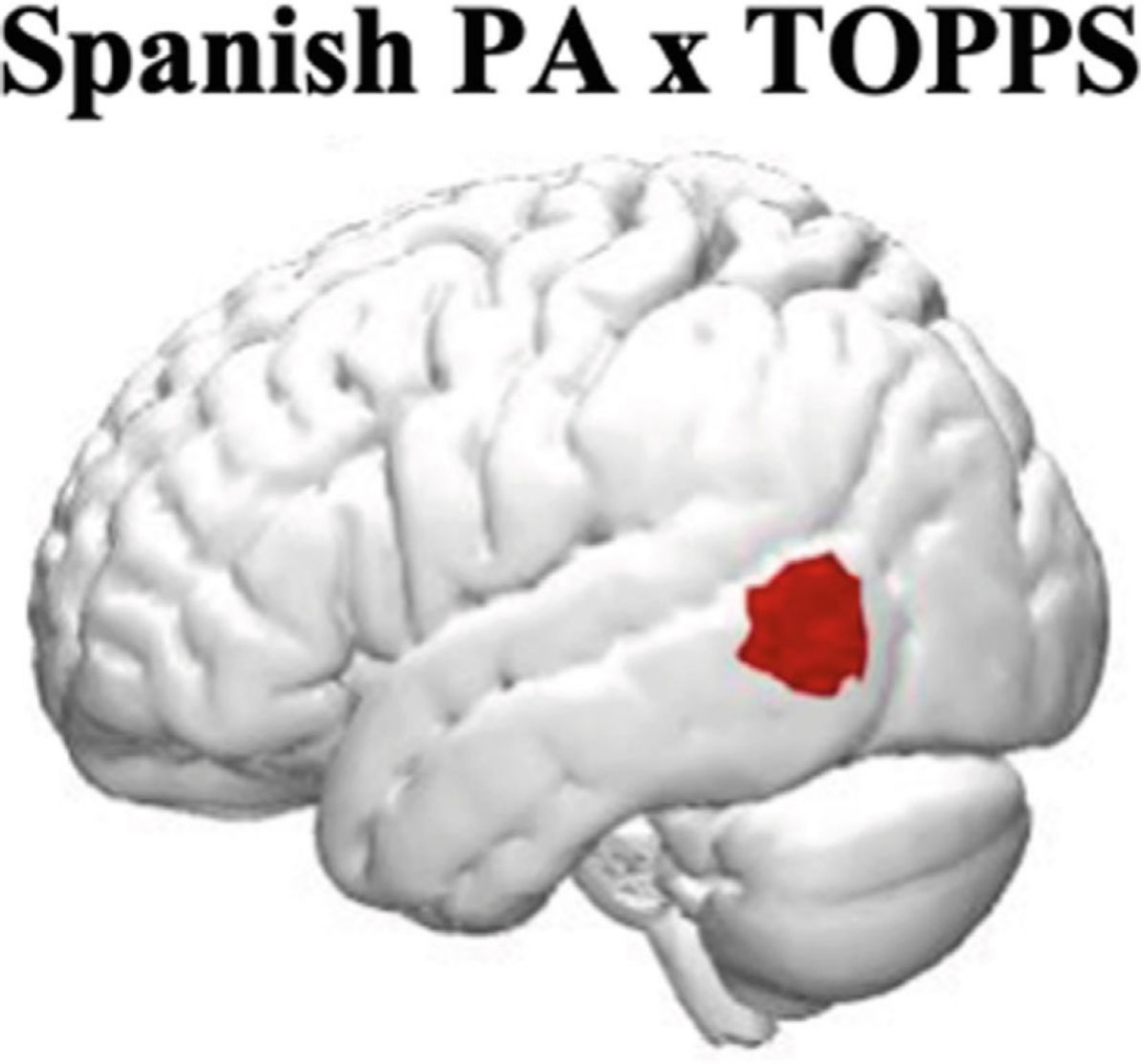
Brain-behavior associations between participant performance on phonology tasks and performance on language and literacy behavioral measures. Only brain-behavior association in Spanish reached significance at FDR corrected level of *q* < .05.

As can be seen in Figure 3, brain-behavior correlations revealed a positive association between Spanish task and left MTG activation in Spanish (FDR corrected level of *q* < .05). No other analyses reached significance.

### GIMME

**Fig. 4.**
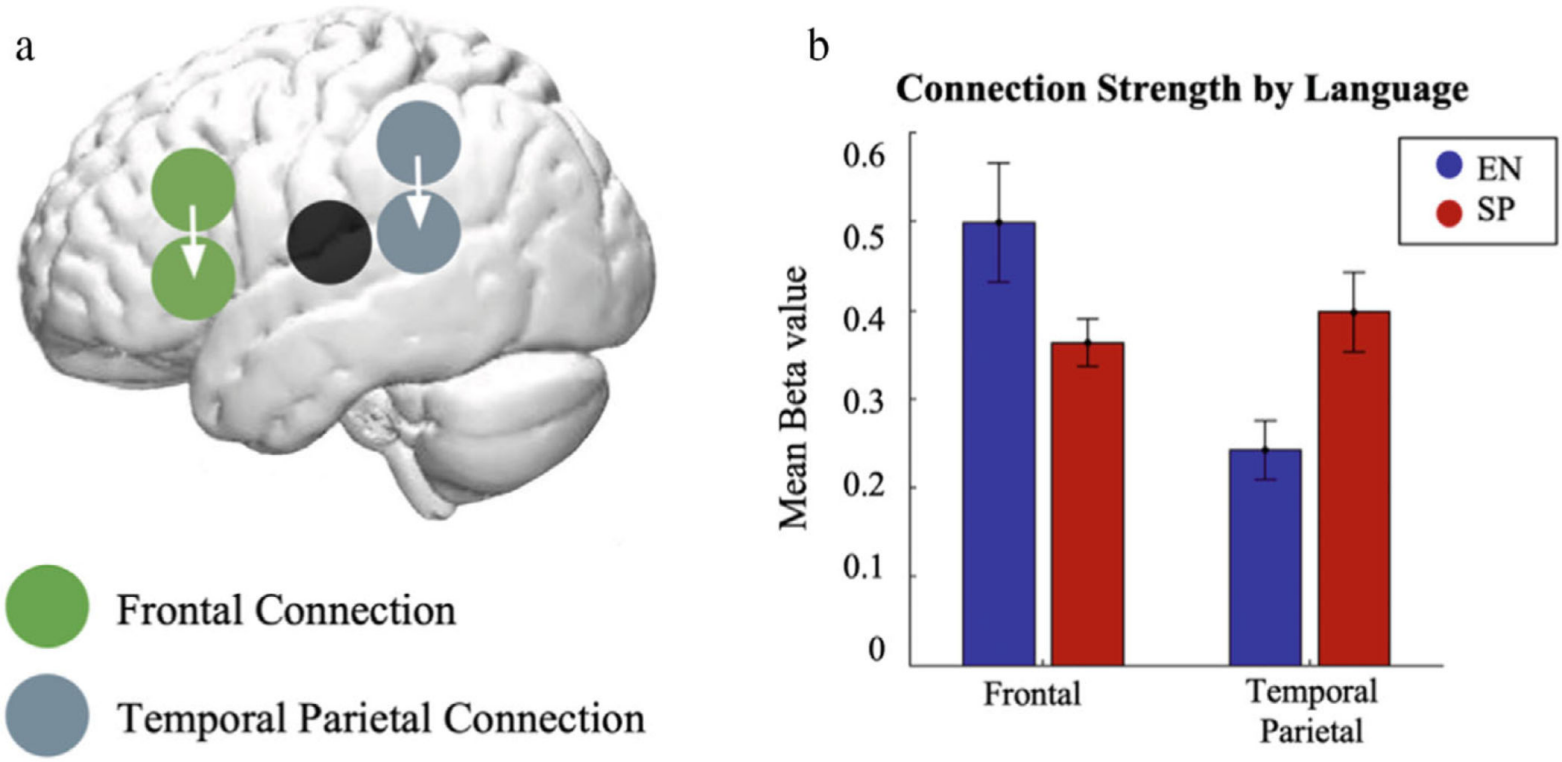
GIMME group-level connections. (a) Group-level connections shown within left frontal regions (2 and 1) and left temporal parietal regions (5 and 4) (b) Connection Strength by Language.

All GIMME maps fit the data well according to averaged indices: RMSEA = .084, SRMR = .029, NNFI = .956, and CFI = .976. GIMME identified two group-level connections, a frontal connection and a temporoparietal connection, which were common across all participants and both languages (Figure 4a). A mixed effect ANOVA revealed that the strengths of these connections varied in the two languages, such that during the English phonology task, the frontal connection was stronger than the temporal connection, while during the Spanish phonology task the temporal connection was stronger than the frontal one (Figure 4b; significant interaction between language (English, Spanish) and strength of connections (frontal, temporoparietal), *F*(1,101) = 11.816, *p* < .001).

In addition to group-level connections, GIMME also identified single participants’ connection maps in each language. None of these other connections was more common for English or Spanish. For most participants (N = 23), the maps estimated during the English and Spanish tasks included the same connections. For the remaining three subjects, only one connection differed between the two languages. However, while there was a high degree of overlap across the two languages *within* participants, there was variability *between* participants, such that up to 12 participants had any given connection in their map. The exploration of the variability between participants exceeds the scope of this study but provides grounds for future work.

## DISCUSSION

The present study offers one of the first systematic and multimethod investigations into the neurocognitive bases of PA in bilingual heritage language speakers. Bilingual Spanish–English heritage language speakers with English-dominant education and varied amounts of heritage language literacy instruction in the US completed PA neuroimaging tasks in each of their languages. Consistent with the idea that PA is a shared cognitive construct in the bilingual mind, the task engaged similar brain regions and revealed some common patterns of functional connectivity across Spanish and English. Nevertheless, the findings also revealed language-specificity: in Spanish, the children showed more widespread activation and stronger functional connectivity in the temporal regions. In contrast, frontal functional connectivity was stronger in the English language. Below we discuss these neurocognitive findings in relation to theories of brain, literacy, and bilingual development.

The bilingual children in this study had age-appropriate language and literacy skills in each of their languages, although there were some significant but still relatively slight differences in performance across tasks (Table 1). In light of the high-dual-language performance, the similarities in patterns of brain activation are logical. In Spanish and in English, children showed activation in left IFG and temporoparietal regions classically associated with phonological analyses (Martin et al., 2015). These frontal and temporal areas of the left-lateralized perisylvian neural network are generally considered essential for the processing of phonological tasks (Hickok & Poeppel, 2016) and so these findings are generally consistent with previous monolingual findings and also suggest a language-common neural base for bilinguals’ two languages.

A direct cross-linguistic comparison revealed stronger bilateral temporal activations in Spanish relative to English, occipitotemporal on the left, and temporoparietal on the right. (Figure 1). These results agree with previous neuroimaging studies that have consistently reported bilateral frontotemporal activation for auditory phonological awareness tasks (e.g. Kovelman et al., 2012), as well as stronger temporal lobe activation when reading in transparent orthographies compared with English (Pauelsu, 2000). In this study, the stronger temporal lobes activation during the task in Spanish may be because of a more automatic recruitment of phonological processing in Spanish compared with English. Indeed, phonological skills were positively associated with left posterior middle temporal lobe activation in Spanish but not in English (Figure 2).

Another interpretation is the unbalanced literacy experiences and proficiency across the two languages. Prior works have suggested a developmental transition from temporal to frontal regions in processing phonological literacy tasks across ages and proficiency levels (meta-analyses by Martin et al., 2015). A similar temporal-to-frontal effect was found by Enge et al. (2020) (also a meta-analyses), who considered a broader variety of auditory language tasks. These prior works have attributed this neurodevelopmental trend to the finetuning of analytical processes that support sound-to-print mappings (Martin et al., 2015) as well as the general advancement of children’s ability to analyze the structural elements of language forms (phonology, syntax, morphology; Enge et al., 2020). It is, therefore, possible that the stronger bilateral temporal activation in Spanish relates to the fact that children’s literacy experiences with Spanish are more informal and home-based and less analytically demanding in contrast to their academic experiences with English.

To understand our findings in a broader cross-linguistic context, let us consider more distant language pairs such as English and Chinese. For instance, in Cao et al. (2021) examined Chinese-English bilinguals with and without dyslexia learning, who were all learning English at school as their L2. Researchers found that both groups exhibited stronger right frontal and right occipital activation in Chinese relative to English during a visual rhyme task. These findings are logical as relative to English, Chinese orthography is more phonologically opaque and more visually demanding—the observed findings thus likely reflect these cross-linguistic differences. Taking the present and Cao et al. (2021) observations, we can suggest that across both formal second language learning contexts and semiformal heritage language learning contexts, emerging readers develop language-specific processes that show the differences between the two languages as they are structured, consistent with recent findings by Cao et al. (2021).

The functional connectivity analyses also suggested similarities and cross-linguistic differences. There were two major connectivity patterns that were common across the two languages: one between the two frontal regions and one between the posterior temporal and an adjacent inferior parietal region, all in the left hemisphere. Cross-linguistic differences were also observed as there was a significant interaction in the strength of the connections across the two languages: in English the frontal connection was stronger and the temporal connection was weaker than in Spanish. As can be seen in Figure 4, whereas the English connections are different between the two regions and different in relation to Spanish, in Spanish, the connections are relatively similar across the two regions.

Both the cross-linguistically common and distinct patterns of functional connectivity are generally consistent with our GLM findings of the patterns of task-modulated activation. In particular, our GLM findings revealed similar patterns of activation in frontal and temporoparietal regions for both languages. The GIMME findings similarly reveal the frontal and temporoparietal patterns of connectivity. These findings are generally consistent with the notions that these two networks are critical to phonological literacy tasks (Martin et al., 2015) and that in the bilingual brain PA is likely to be supported by a common underlying neurocognitive system (Chung et al., 2019). The distinct connectivity patterns also echo our GLM findings of stronger temporal activation in Spanish than in English and significant brain-behavior correlations in the left temporal lobe in Spanish only.

To sum our observations and interpretations, the academic pressure for analyzing words in an English academic context may heighten the functionality of higher-order linguistic processing typically supported by the left IFG regions—as revealed by stronger frontal connectivity in English (Hagoort, 2019). In contrast, experiences of speaking and reading in Spanish that are more casual might be effective in supporting heritage language maintenance and growth but are entraining different neural paths that are associated with more automated and potentially more meaning-based processes as revealed by stronger temporal activations, functional connectivity, and brain-behavioral correlations with left MTG, a region typically associated with semantic processes.

Present findings help inform theoretical perspectives on literacy development and neural plasticity in relation to bilingualism and literacy development processes. Prior works have shown that dual-language experiences yield both differences and similarities in how two languages are organized within the bilingual brain, and our work contributes to a better understanding of the nature of these processes. For instance, Brignoni-Perez et al. (2020, 2022) found engagement of similar brain regions for both languages within a population of adult bilinguals with 12 years of systematic dual-language and literacy experience and high dual-language reading proficiency. Notably, opportunities are often limited for many heritage language speakers to receive formal literacy instruction in their heritage language. Our findings reveal that even young readers with English-dominant instruction show a similar pattern of neural engagement, recruiting similar regions while processing each of their languages. The ICM predicts similarities for shared tasks, such as phonological awareness skills, but it also predicts specificity depending on the features of a given language. We knew that was the case with adults. Still, now we show that even with English (society’s dominant language in the US) being a dominant language of literacy instruction, children develop both common and language-specific literacy skills as previously reported in bilingual and monolingual adults with abundant literacy experience in each of their languages, addressing the value of encouraging heritage language instruction across all contexts.

## CONCLUSIONS

Our study is one of the first to explore the neurocognitive basis for phonological awareness in a population of heritage language Spanish–English bilingual children, an immigrant ethnic and language minority population underrepresented in neuroimaging research. Using fNIRS, a child friendly, non-invasive neuroimaging technique that we have adapted for use with individuals of varied hair color and texture, our findings shed light on the neurobiological and experiential factors that guide bilingual development. Taken together, the findings suggest that literacy skills developed in each of the bilinguals’ languages have a cumulative effect on emergent literacy as bilingualism facilitates language-common mechanisms of reading development, thus arguing in favor of heritage language maintenance and literacy instruction.

## Supplementary Material

Data S1 and Appendixes 1 and 2

## Figures and Tables

**Table 1:** Behavioral And Neuroimaging Task Performance

		*English*	*Spanish*

Oral language measures	Vocabulary (ss)	100.8 (19.6)*	109.0 (17.9)*
	Phonological awareness (ss)	11.1 (3.5)***	13.7 (6.3)***
Literacy measures	Single word reading (ss)	110.1 (17.6)	110.1 (27.60)
	Passage comprehension (ss)	98.9 (14.6)***	93.3 (16.4)***
fNIRS phonology task	Accuracy (%)	76.9 (8.8)*	70.4 (16.6)*
	Response time (ms)	1,525.1 (244.5)	1,629.3 (343.4)

*Note*. Reported values are mean (standard deviation). Comparison between languages was assessed with a *t*-test. ss = standard score.

∗ *p* < .05.

∗∗∗ *p* < .001.

**Table 2:** Brain Activation During Phonological Task Relative to Rest (β-Values for Channels that were Significantly Active at *p* < .01, and Estimates of Brain Regions and Corresponding Montreal Neurological Institute (MNI) Coordinates for These Active Channels Based on Hu et al., 2020)

				*MNI Coordinates*		
	
*Hemisphere*	*Channel*	*Region*	*β*	x	y	z

*English PA*						
L	1	vIFG, MFG	7.140	−50	50	−17
L	2	vIFG, Precentral	5.739	−62	29	−14
L	5	Precentral, STG, IFG	5.294	−65	12	−11
L	7	Postcentral, STG, Precentral	5.495	−68	−4	−9
L	10	STG, Postcentral, IPL	5.812	−64	−16	14
Spanish PA						
L	1	vIFG, MFG	6.540	−50	50	−17
L	3	vIFG, Precentral	6.198	−62	29	−14
L	10	STG, Postcentral, IPL	5.947	−68	−4	−9
R	20	MTG, STG, MOG, ITG	6.587	55	50	21
Spanish > English						
L	21	MOG, ITG, FG, MTG	11.984	−51	−54	−38
R	37	IPL, SMG, AG	9.621	53	43	−17
R	39	AG, Prenucleus, IPL, STG	−10.579	45	53	−15
R	40	MOG, MTG, ITG	7.664	45	63	18

## Data Availability

The data that support the findings of this study are openly available in DeepBlue at https://deepblue.lib.umich.edu/data/concern/data_sets/6969z110z, reference number https://doi.org/10.7302/kxgf-ps11.
