# Supplementary material for: What’s in the Sound? Common and Language-Specific Patterns in Brain Activation and Functional Connectivity for Phonological Awareness in Spanish–English Bilinguals
Source: Mind Brain Educ. Author manuscript; Available in PMC 2025 Aug 5. (PMC12323680; doi:10.1111/mbe.12410)
Supplement: Data S1 and Appendixes 1 and 2 [file NIHMS2033402-supplement-Data_S1_and_Appendixes_1_and_2.pdf]

APPENDIX 1

Appendix Table 1
Estimated Left Hemisphere Brain Regions Covered by the fNIRS Probeset.

Channel	*Region*	*MNI coordinates*		
		x	*y*	*z*
1	vIFG, MFG	−50	50	−17
2	MFG, dIFG	−53	49	−1
3	vIFG, Precentral	−62	29	−14
4	dIFG, MFG	−59	33	5
5	Precentral, STG, IFG	−65	12	−11
6	IFG, Precentral, MFG	−62	17	8
7	Postcentral, STG, Precentral	−68	−4	−9
8	Precentral, Postcentral	−64	−1	11
9	STG, Postcentral, IPL, TTG	−67	−19	−6
10	Precentral, Postcentral, IPL	−64	−16	14
11	STG, SMG, IPL, Postcentral	−63	−33	−3
12	IPL, Postcentral, SMG	−60	−30	16
13	SMG, STG, MTG, IPL	−56	−46	−2
14	IPL, SMG, AG	−53	−43	17
15	MTG, AG, STG, SMG	−46	−59	1
16	AG, Prenucleus, IPL, STG	−45	−53	15
17	MTG, STG	−67	−22	−26
18	MTG, STG	−63	−36	−23
19	ITG, MTG, FG	−58	−40	−40
20	MTG, STG, MOG, ITG	−55	−50	−21
21	MOG, ITG, FG, MTG	−51	−54	−38
22	MOG, MTG, ITG	−45	−63	−18
23	ITG, IOG, MOG, FG	−44	−64	−34

Appendix Table 2
Descriptive Data for GIMME Participants ($N = 26$)

	English	*Spanish*
Vocabulary (ss)	101.1 (18.5)	109.2 (18.4)
Single Word Reading (ss)	106.4 (18.9)	104.9 (32.9)
fNIRS phonology task accuracy (%)	77.9 (8.0)*	69.6 (15.5)*
fNIRS phonology task response time (ms)	1,553.7 (250.3)	1,602.8 (341.6)

Note. Reported values are mean (standard deviation). Comparison between languages was assessed with a t-test. ss = standard score.
*$p < 0.05$.
